# Supplementary material for: Binding mechanism of the matrix domain of HIV-1 gag on lipid membranes
Source: eLife. 2020 Aug 18;9:e58621. doi: 10.7554/eLife.58621 (PMC7476761; doi:10.7554/eLife.58621)
Supplement: Transparent reporting form [file elife-58621-transrepform.pdf]

Westbrook Centre, Milton Road
Cambridge CB4 1YG
UK

P 01223 855340
W elifesciences.org
T @elife

eLife's transparent reporting form

We encourage authors to provide detailed information *within their submission* to facilitate the interpretation and replication of experiments. Authors can upload supporting documentation to indicate the use of appropriate reporting guidelines for health-related research (see EQUATOR Network), life science research (see the BioSharing Information Resource), or the ARRIVE guidelines for reporting work involving animal research. Where applicable, authors should refer to any relevant reporting standards documents in this form.

If you have any questions, please consult our Journal Policies and/or contact us: editorial@elifesciences.org.

Sample-size estimation

- You should state whether an appropriate sample size was computed when the study was being designed
- You should state the statistical method of sample size computation and any required assumptions
- If no explicit power analysis was used, you should describe how you decided what sample (replicate) size (number) to use

Please outline where this information can be found within the submission (e.g., sections or figure legends), or explain why this information doesn't apply to your submission:

This section does not apply to the theoretical technique we employ for our studies, molecular dynamics simulation. We discuss our statistical analysis approach and simulation replicates in following sections. The membrane model selection and protein structure used in this study are described in the Methods section in the main manuscript, and Table 1 in the *Results* section. Figure 2 – Figure Supplement 1 shows the chemical structures of the lipids used in this study, selected based on a published experimental study examining protein dynamics of the same system (HIV-1 Gag assembly).

Replicates

- You should report how often each experiment was performed
- You should include a definition of biological versus technical replication
- The data obtained should be provided and sufficient information should be provided to indicate the number of independent biological and/or technical replicates
- If you encountered any outliers, you should describe how these were handled
- Criteria for exclusion/inclusion of data should be clearly stated
- High-throughput sequence data should be uploaded before submission, with a private link for reviewers provided (these are available from both GEO and ArrayExpress)

Please outline where this information can be found within the submission (e.g., sections or figure legends), or explain why this information doesn't apply to your submission:

eLife Sciences Publications, Ltd is a limited liability non-profit non-stock corporation incorporated in the State of Delaware, USA, with company number 5030732, and is registered in the UK with company number FC030576 and branch number BR015634 at the address Westbrook Centre, Milton Road Cambridge CB4 1YG, UK | March 2019

We described the systems, size, and number of simulation runs per system under the *Methods* section as well as in Table 1 in the main manuscript and Table S2 in Supplementary File 1.

As standard practice, MD simulation trajectories are run in pairs or triplicates to ensure independent trajectories for posterior statistical analysis. We run two independent trajectories for the short protein-membrane interactions, as listed on Table 1. Three replicates were run for the microsecond simulations that explore insertion of the lipidated tail of the protein into the membrane; and two for the membrane systems with three protein units on the surface, as listed on Table S2.

Statistical reporting

- Statistical analysis methods should be described and justified
- Raw data should be presented in figures whenever informative to do so (typically when N per group is less than 10)
- For each experiment, you should identify the statistical tests used, exact values of N, definitions of center, methods of multiple test correction, and dispersion and precision measures (e.g., mean, median, SD, SEM, confidence intervals; and, for the major substantive results, a measure of effect size (e.g., Pearson's r, Cohen's d)
- Report exact p-values wherever possible alongside the summary statistics and 95% confidence intervals. These should be reported for all key questions and not only when the p-value is less than 0.05.

Please outline where this information can be found within the submission (e.g., sections or figure legends), or explain why this information doesn't apply to your submission:

The data analysis as described in detail on Table S3, was computed over equilibrated sections of the simulation trajectories, unless the analysis shows the time evolution of a given property. Values reported in this manuscript were blocked averaged every 10ns over the last 100-300ns of trajectory, and the standard error of the measurement reported along with the average. In the case of timeseries shown in the figures, we show the raw data in a faded hue, while the moving average is shown in bold colors and computed using 5ns blocks.

(For large datasets, or papers with a very large number of statistical tests, you may upload a single table file with tests, Ns, etc., with reference to sections in the manuscript.)

Group allocation

- Indicate how samples were allocated into experimental groups (in the case of clinical studies, please specify allocation to treatment method); if randomization was used, please also state if restricted randomization was applied
- Indicate if masking was used during group allocation, data collection and/or data analysis

Please outline where this information can be found within the submission (e.g., sections or figure legends), or explain why this information doesn't apply to your submission:

This does not apply to a molecular dynamics simulation study; this theoretical approach generates a trajectory for all the atoms in a simulation box based on the forces that act on them. The predicted trajectory reproduces the natural interaction among the components of the system based on statistical thermodynamics, and posterior analysis allows us to compute distances, forces, and angles between atoms or groups of atoms. Similarly, we can extract mechanical and structural data of our system of study based on time averages of the simulation trajectory and across replicate runs of a given system.

Additional data files ("source data")

- We encourage you to upload relevant additional data files, such as numerical data that are represented as a graph in a figure, or as a summary table
- Where provided, these should be in the most useful format, and they can be uploaded as "Source data" files linked to a main figure or table
- Include model definition files including the full list of parameters used
- Include code used for data analysis (e.g., R, MatLab)
- Avoid stating that data files are "available upon request"

Please indicate the figures or tables for which source data files have been provided:

We include the following source files:

- Data to generate the lipid density plots in Figures 2, Figure 2 – Figure Supplement 2, Figure 5 – Figure Supplement 1 & 2. Namely, the xy coordinates of a given lipid species averaged over the last 50ns or 200ns of trajectory depending on the system (stated on the corresponding figure caption, and on Table S3.

- Data to generate the histograms and heatmaps in Figure 4; the time series of the distance from the bilayer for the first and last carbons of the protein's lipidated tail.

- Data of the projected trajectories onto the (2) slowest independent components identify by tICA to generate the heatmaps in Figures 7 and Figure 7 – Figure Supplement 1. Each data file contains the full data set of tICs identified from this analysis (10 tICs per system)

These plots were generated using *Pandas, Matplotlib* and *Seaborn* data visualization libraries in Python. Scripts for the corresponding figures are also provided). A legend for each file is included in the main manuscript.
